# Bibliometric analysis of stereotactic ablative radiotherapy for oligometastases

**DOI:** 10.3389/fmed.2026.1782986

**Published:** 2026-03-18

**Authors:** Yupeng Di, Zhuo Song, Yingjie Wang, Lingling Meng, Jing Li

**Affiliations:** 1 Department of Radiation Protection Medicine, School of Preventive Medicine, Fourth Military Medical University, Xi’an, China; 2 Ministry of Education Key Lab of Hazard Assessment and Control in Special Operational Environment, Xi’an, China; 3 Department of Radiation Oncology, Air Force Medical Center, PLA, Beijing, China; 4 Department of Radiation Oncology, Peking University Shougang Hospital, Beijing, China; 5 Department of Radiation Oncology, Senior Department of Oncology, The First Medical Center of PLA General Hospital, Beijing, China

**Keywords:** bibliometric analysis, cancer, oligometastases, radiation oncology, SABR, stereotactic ablative radiotherapy

## Abstract

**Background:**

Oligometastases, an intermediate stage of metastatic cancer, are increasingly managed with Stereotactic Ablative Radiotherapy (SABR), a highly precise local therapy representing a pivotal paradigm shift for improving patient outcomes. This study aimed to comprehensively map the research landscape of SABR for oligometastases.

**Methods:**

We conducted a cross-sectional bibliometric analysis of 1,066 publications from the Web of Science Core Collection and PubMed from January 1, 2006, to December 31, 2025. Publication trends, key contributors, and collaboration networks were quantified and visualized using bibliometrix, VOSviewer, and CiteSpace.

**Results:**

Our results indicate significant and consistent growth in this field since 2006, with the United States, Italy, and Canada as leading countries, and Humanitas University among the most productive institutions, alongside key authors like Marta Scorsetti. Influential research appeared in leading oncology journals, and keyword analysis identified hotspots in cancer types, metastatic sites, treatment strategies, and the integration of immunotherapy and AI. Landmark clinical trials (e.g., SABR-COMET, ORIOLE) were highly cited.

**Conclusion:**

This comprehensive overview underscores the rapid development, international collaboration, and critical research directions within the field, highlighting SABR’s translational potential and providing a foundation for identifying future research priorities.

## 1 Introduction

Oligometastases, a state of limited metastatic disease, represent an intermediate phase between localized and widespread metastatic cancer. Globally, a significant proportion of cancer patients will develop metastases during their disease course. Among these, it is estimated that approximately 20 to 50% of patients with advanced disease—depending on the primary tumor type, such as prostate, breast, or non-small cell lung cancer—initially present with a limited number of metastatic lesions, characterizing the oligometastatic state. This widespread global incidence highlights a crucial clinical challenge and emphasizes the need for specialized management strategies. This concept challenges the traditional dogma that metastatic disease is uniformly incurable, prompting the exploration of aggressive local therapies in conjunction with systemic treatments (1). Stereotactic ablative radiotherapy (SABR), also known as stereotactic body radiation therapy (SBRT), is a highly precise form of radiation therapy that delivers high doses to extracranial tumors with sub-millimeter accuracy, typically in a few fractions (2, 3). Owing to its ability to achieve high rates of local control with minimal toxicity, SABR is positioned as a promising therapeutic modality for patients with oligometastases (4–6). The application of SABR in this setting signifies a paradigm shift in cancer management, offering the potential for improved progression-free survival, overall survival, and even cure in select patients. This transformation is comprehensively illustrated in Supplementary Figure 1.

The rapid evolution and increasing adoption of SABR for oligometastases necessitate a comprehensive understanding of its research landscape. Bibliometric analysis serves as a powerful quantitative tool to map the intellectual structure, identify emerging trends, and evaluate the impact of scientific research within a specific field. By analyzing publication patterns, authorship, institutional collaborations, and citation metrics, bibliometric studies provide invaluable insights into the growth, key players, and thematic development of a research area.

This study aims to provide a comprehensive bibliometric analysis of publications related to SABR for oligometastases, utilizing data extracted from the Web of Science Core Collection (WoSCC) and PubMed up to December 31, 2025. This analysis elucidates publication trends, leading countries, institutions, and authors, as well as influential journals and highly cited articles. Furthermore, it explores the conceptual evolution and key advances in the field, identifies challenges, and discusses future perspectives such as the integration of Artificial Intelligence (AI). The findings will not only enhance our understanding of the current status of SABR research but also provide valuable insights to guide future investigations and clinical applications.

## 2 Materials and methods

### 2.1 Search strategy and data collection

**Figure 1:**
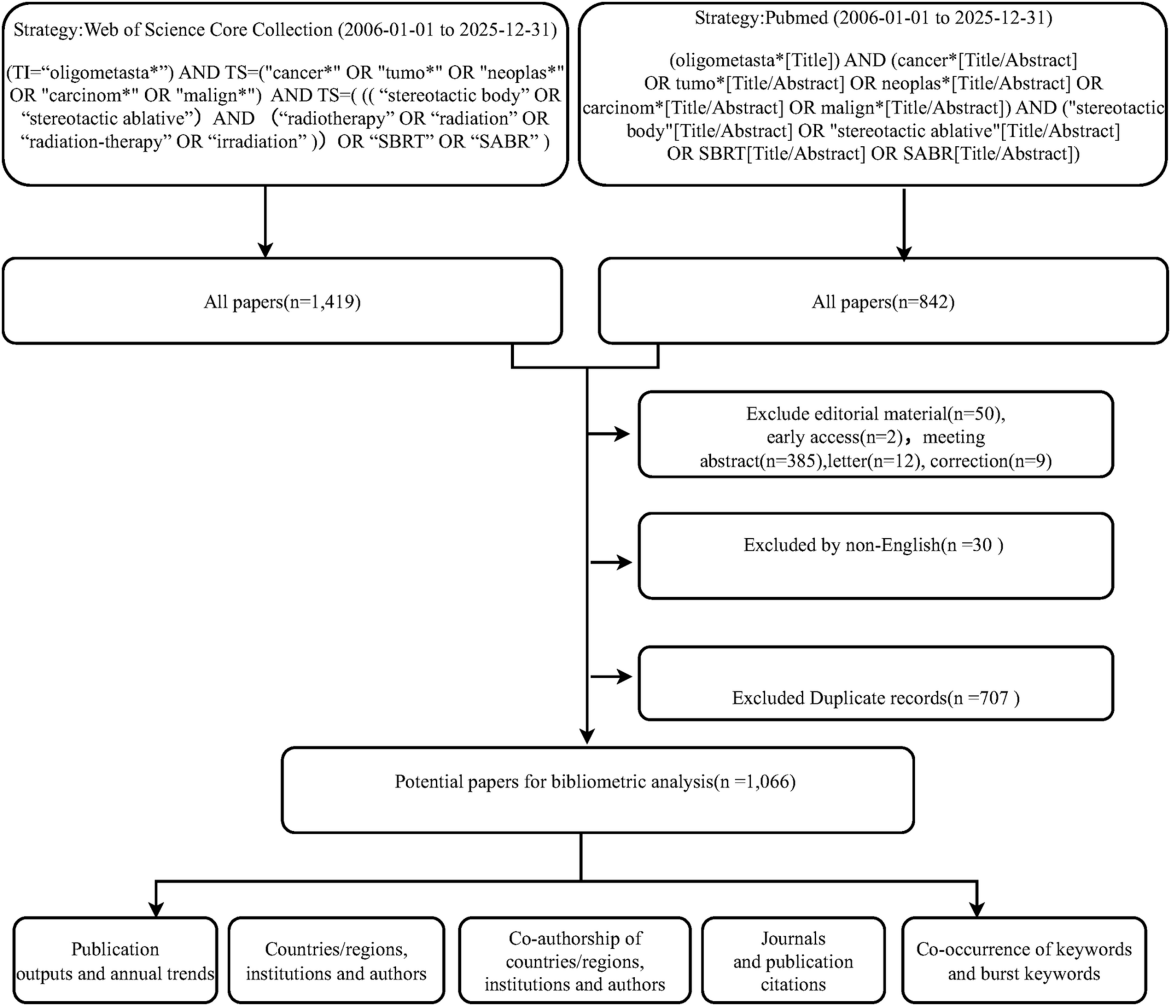
The data collection and retrieval strategy flow chart based on Web of Science and PubMed databases (2006–2025).

This cross-sectional study analyzed publications spanning the period from January 1, 2006, to December 31, 2025. The exact literature search and data download were concurrently conducted on January 5, 2026. To ensure comprehensive and high-quality coverage of the literature, both the Web of Science Core Collection (WoSCC) and PubMed databases were utilized as data sources. For WoSCC, the Science Citation Index Expanded (SCI-EXPANDED) and Social Sciences Citation Index (SSCI) indices were used, and no restrictions were applied to the citation indexes. To avoid analytical bias and ensure data integrity, a rigorous screening and deduplication process was performed. The detailed process of data collection and retrieval is illustrated in Figure 1 and further detailed in a PRISMA-style flow diagram (Supplementary Figure 2). The retrieved publications met the following criteria.

First, specific search strings were designed (fully executable search strings are provided in Supplementary File 1). For WoSCC, the query was: (TI = “oligometasta*”) AND TS = (“cancer*” OR “tumo*” OR “neoplas*” OR “carcinom*” OR “malign*”) AND TS = (((“stereotactic body” OR “stereotactic ablative”) AND (“radiotherapy” OR “radiation” OR “radiation-therapy” OR “irradiation”)) OR “SBRT” OR “SABR”). The restriction of the term “oligometasta*” explicitly to the Title field was intentionally applied to precisely capture studies where the oligometastatic state is the central focus, thereby minimizing false-positive results from casual mentions in abstracts. For PubMed, the query similarly restricted “oligometasta*” to Title.

Second, the timeframe was strictly limited to publications from January 1, 2006, to December 31, 2025. Third, regarding document types in WoSCC, meeting abstracts, editorial materials, letters, corrections, and early access records were excluded. Fourth, only articles published in English were included. Finally, following database integration, a rigorous deduplication algorithm was applied using EndNote software; duplicates were removed matching primary Digital Object Identifiers (DOIs), followed by exact title and author matching. Any remaining fuzzy conflicts were resolved manually. Following the removal of 707 duplicate records, a total of 1,066 valid records were ultimately included.

### 2.2 Data analysis and visualization

The R package *bibliometrix* (v4.4.1) was used to quantify the number of publications, journals, and local citations (explicitly defined here as citations strictly occurring within the analyzed 1,066-record dataset, distinguishing them from total global database citations) (7–9). VOSviewer software (version 1.6.17) was utilized to create visualized network maps by extracting bibliographic information on authors, institutions, citations, and keywords, detailing their interrelationships (10). *CiteSpace* (v6.1.R6) was applied to detect keyword citation bursts (11, 12). Journal Impact Factors (IF) were obtained from the 2024 Journal Citation Reports to ensure chronological consistency across tables.

### 2.3 Research ethics

All data were obtained and downloaded from publicly available databases (WoSCC and PubMed). The study did not interact with human subjects or animals, and no patient-level or identifiable data were used; thus, ethical approval was not required.

## 3 Results

### 3.1 Analysis of publication outputs and annual publication trends

**Figure 2:**
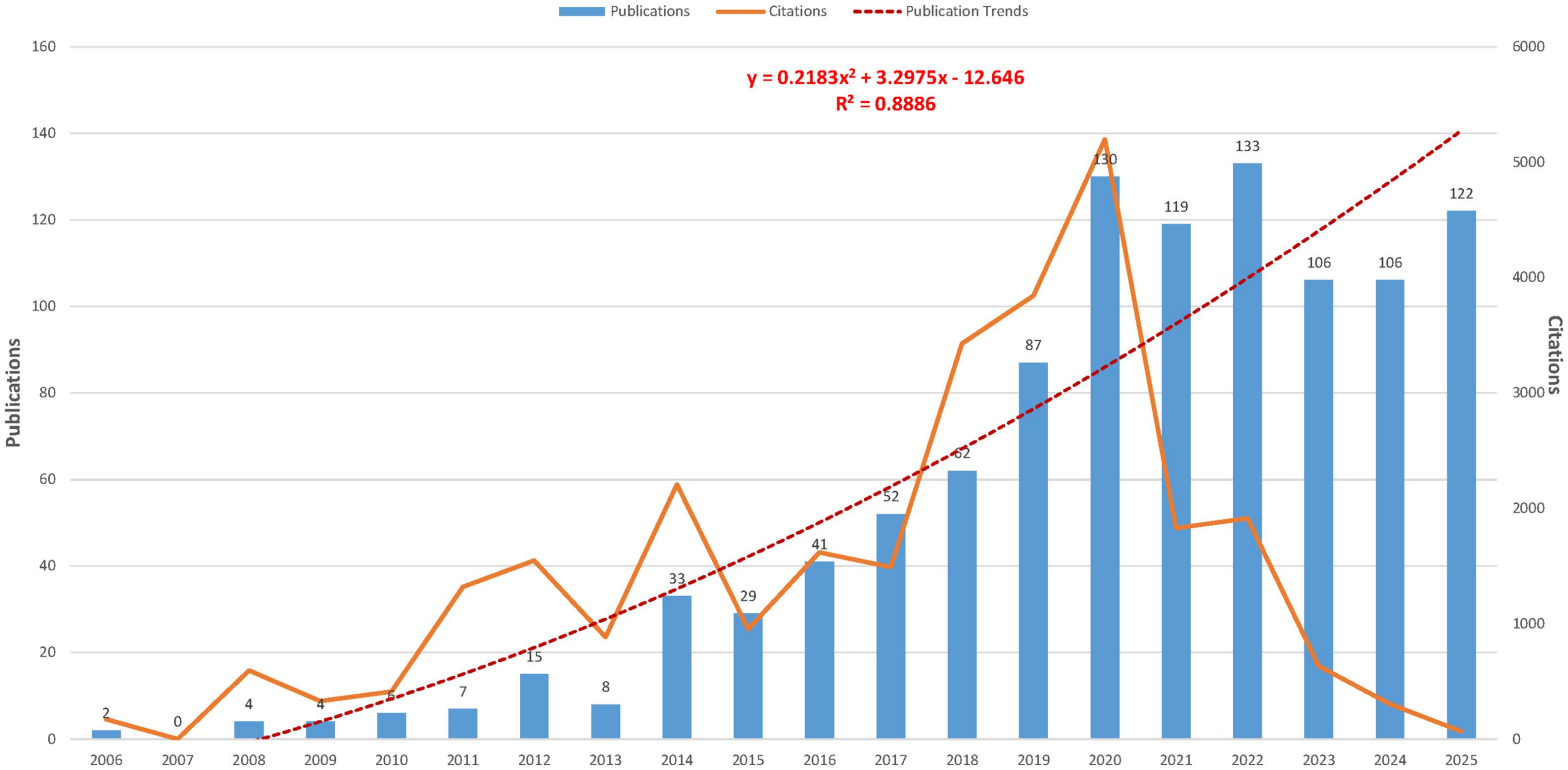
Annual publication and citation trends regarding SABR for oligometastases research (2006–2025).

In total, 1,066 unique publications related to SABR for oligometastases were retrieved from WoSCC and PubMed up to December 31, 2025. The annual publication trends, along with their citations, are depicted in Figure 2. Research in this domain has exhibited remarkable and consistent growth since 2006. The number of publications has steadily increased, peaking significantly in recent years. The annual publication count demonstrates a strong upward trajectory. The trend is well-described by a quadratic equation: y = 0.2183x^2^ + 3.2975x − 12.646, with a high coefficient of determination (R^2^ = 0.8886), signifying a robust growth pattern.

### 3.2 Distribution of countries/regions, institutions and authors

**Figure 3:**
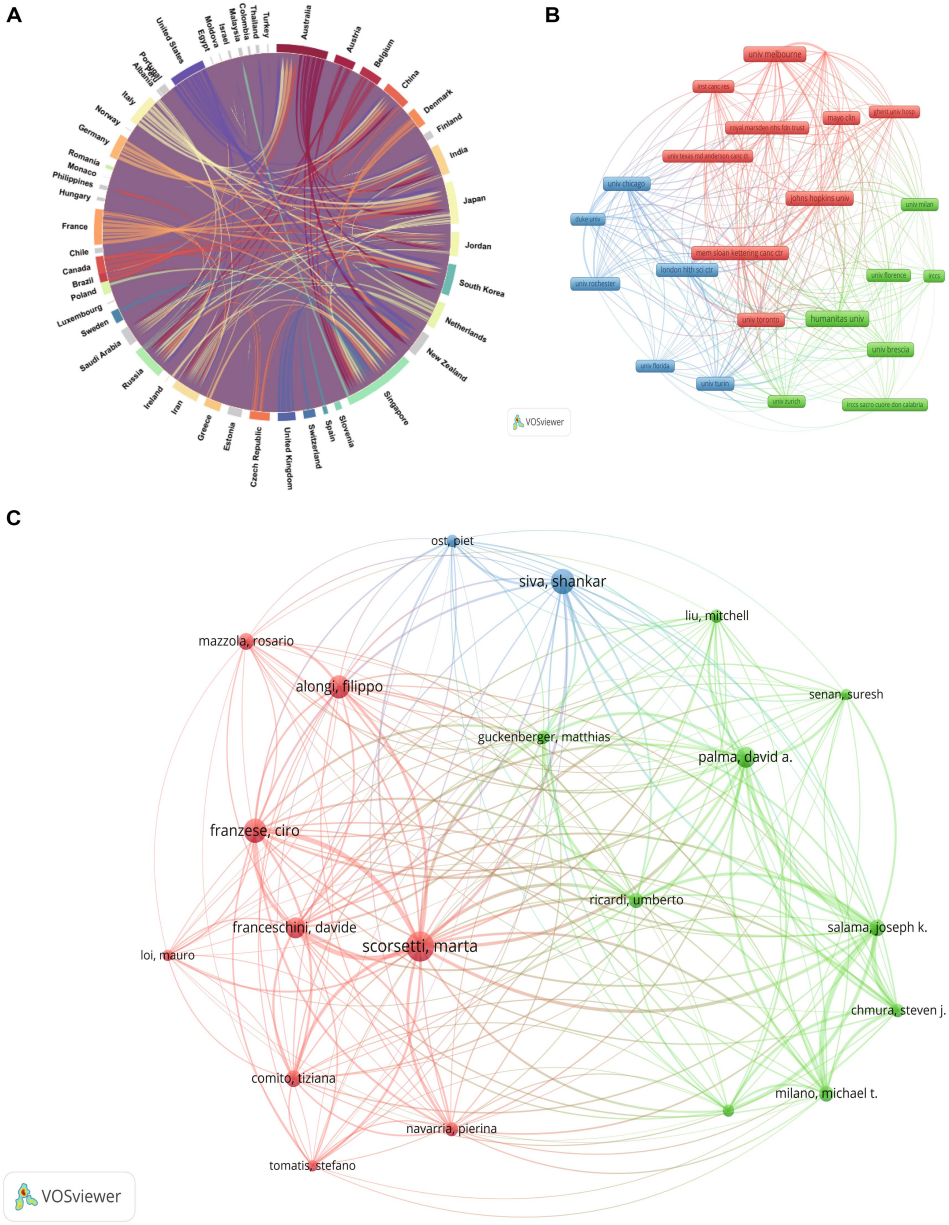
Co-authorship network of countries/regions **(A)**, institutions **(B)**, and authors **(C)**. **(A)** The USA (blue) acts as a central node with extensive connections, indicating its significant role in collaborations. Italy (yellow) forms a prominent collaborative cluster, and Canada (orange-red) also demonstrates strong connectivity. **(B)** Among 23 institutions, 3 clusters were identified. Cluster 1 includes major research institutions such as Johns Hopkins University and the University of Melbourne. Cluster 2 is primarily composed of Italian institutions like Humanitas University and the University of Brescia. Cluster 3 features institutions such as Duke University and the University of Chicago. **(C)** Key authors such as Marta Scorsetti, Shankar Siva, and David A. Palma are prominent contributors in this field.

A total of 59 countries/regions contributed to the publications in the current study (Table 1). The United States submitted the highest volume of publications (*n* = 230, 21.6%), followed by Italy (*n* = 140, 13.1%), and Canada (*n* = 69, 6.5%). The strong collaborative networks among these countries are visualized in Figure 3A, where the USA acts as a central, extensively connected node; Italy forms a prominent cluster; and Canada also demonstrates strong connectivity.

**Table 1:** The top 10 productive countries/regions.

Rank	Country/Region	Publications	Publications (%)	Citations
1	United States	230	21.6	9,304
2	Italy	140	13.1	2,816
3	Canada	69	6.5	4,929
4	China	57	5.3	594
5	Germany	43	4.0	901
6	Japan	43	4.0	926
7	France	38	3.6	510
8	United Kingdom	36	3.4	1,110
9	Australia	35	3.3	1,303
10	Belgium	29	2.7	3,157

These publications originated from a diverse range of institutions. The top 10 productive institutions are listed in Table 2. Humanitas University (Italy) led with 38 publications. The institutional co-authorship network in Figure 3B categorizes these into 3 clusters: Cluster 1 encompasses major institutions like Johns Hopkins University and the University of Melbourne; Cluster 2 primarily consists of Italian institutions; and Cluster 3 features institutions like Duke University.

**Table 2:** The top 10 productive institutions.

Rank	Institution	Country	Total publications	Citations
1	Humanitas University	Italy	38	675
2	University of Melbourne	Australia	36	1,568
3	Peter MacCallum Cancer Centre	Australia	33	1,392
4	Johns Hopkins University	United States	30	1850
5	University of Brescia	Italy	29	408
6	Memorial Sloan Kettering Cancer Center	United States	28	584
7	University of Toronto	Canada	27	489
8	University of Chicago	United States	26	2,531
9	London Health Sciences Centre	Canada	24	4,183
10	University of Turin	Italy	24	2,305

The top 20 productive authors are listed in Table 3. Marta Scorsetti (Italy) was the most productive author with 36 publications. Shankar Siva (Australia) followed with 30 publications. Notably, David A. Palma (Canada) achieved an exceptionally high average citation count of 165. The co-authorship network of key authors is presented in Figure 3C, highlighting strong research communities and deep cross-institutional collaborations driving advancements in SABR for oligometastases.

**Table 3:** The top 20 productive authors.

Rank	Author	Country	Total publications	Total citations	Average citations	H-index	g-index
1	Marta Scorsetti	Italy	36	2,224	62	20	48
2	Shankar Siva	Australia	30	1,349	45	17	33
3	Ciro Franzese	Italy	29	499	17	15	22
4	Filippo Alongi	Italy	28	786	28	16	30
5	David A. Palma	Canada	25	4,115	165	17	28
6	Davide Franceschini	Italy	25	519	21	14	23
7	Joseph K. Salama	United States	20	1,563	78	16	21
8	Tiziana Comito	Italy	20	526	26	14	23
9	Umberto Ricardi	Italy	19	2,205	116	13	22
10	Michael T. Milano	United States	19	1781	94	14	19
11	Pierina Navarria	Italy	17	549	32	14	22
12	Steven J. Chmura	United States	16	1,138	71	11	16
13	Matthias Guckenberger	Switzerland	16	1,601	100	16	27
14	Mitchell Liu	United States	16	3,234	202	15	19
15	Ralph R. Weichselbaum	United States	15	2,103	140	13	15
16	Piet Ost	Belgium	15	2,818	188	14	21
17	Suresh Senan	Netherlands	14	3,732	267	12	14
18	Simon S. Lo	United States	12	651	54	10	12
19	Phuoc T. Tran	United States	12	497	41	12	19
20	Alexander V. Louie	Canada	11	2,797	254	11	16

### 3.3 The co-occurrence of keywords and burst keyword detection

**Figure 4:**
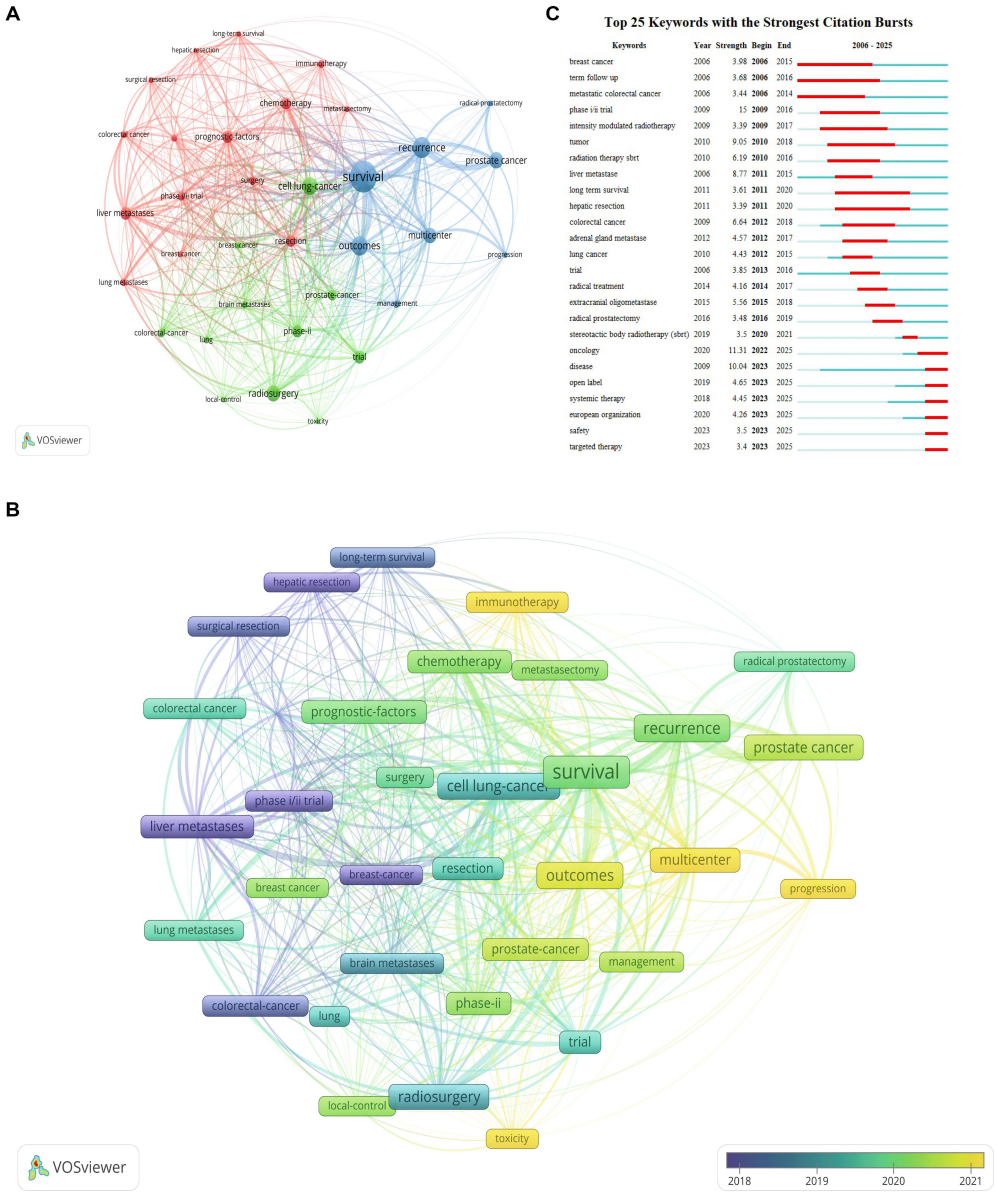
Keyword co-occurrence cluster analysis **(A)** and burst keyword detection **(C)**. **(A)** Three clusters were identified. Cluster 1 (15 items) primarily focuses on cancer types (e.g., breast cancer, colorectal cancer), metastatic sites (e.g., liver metastases, lung metastases), surgical interventions and systemic treatments (e.g., chemotherapy, immunotherapy), long-term survival, clinical trials, and prognostic factors. Cluster 2 (11 items) emphasizes specific cancers (e.g., brain metastases, lung cancer, prostate cancer), local control, radiosurgery, and toxicity. Cluster 3 (8 items) revolves around the management, recurrence, and survival outcomes of prostate cancer. SABR for oligometastases has been a research hotspot in recent years, particularly concerning its combination with different cancer types and treatment strategies. **(B)** Time-overlay network visualization of keywords, indicating the trends and connections over time. **(C)** Emerging research frontiers include the combined application of SABR with immunotherapy, the role of AI in treatment planning and prediction, personalized treatment strategies, and the optimization of SABR for specific metastatic sites (e.g., brain metastases).

Keyword analysis provides insights into the thematic structure and evolution of the field. Figure 4 illustrates a keyword co-occurrence cluster analysis (A) and burst keyword detection (C). Figure 4A identified 3 clusters. Cluster 1 primarily focuses on cancer types (e.g., breast cancer, colorectal cancer), metastatic sites, and systemic treatments (e.g., chemotherapy, immunotherapy). Cluster 2 emphasizes specific cancers (e.g., brain metastases, lung cancer), local control, and radiosurgery. Cluster 3 revolves around the management and survival outcomes of prostate cancer. Furthermore, Figure 4C highlights emerging research frontiers, including the combined application of SABR with immunotherapy, personalized treatment strategies, and the optimization of SABR for specific sites.

### 3.4 Analysis of source journals and publication citations

Numerous journals were involved in publications related to SABR for oligometastases. The top 10 journals are listed in Table 4. The *International Journal of Radiation Oncology Biology Physics* was the most productive journal with 70 articles (6.6%), followed by *Radiotherapy and Oncology* with 44 articles (4.1%), and *Cancers* with 41 articles (3.8%). This indicates that oncology-focused journals are the primary outlets to publish and disseminate research in this domain.

**Table 4:** The top 10 journals published most on research on SABR for oligometastases.

Rank	Source	Category	IF (2024)	Number of articles (percentage)	Total citations
1	*International Journal of Radiation Oncology Biology Physics*	Oncology	6.5	70 (6.6%)	2,569
2	*Radiotherapy and Oncology*	Oncology	5.3	44 (4.1%)	2,140
3	*Cancers*	Oncology	4.4	41 (3.8%)	268
4	*Frontiers in Oncology*	Oncology	3.5	29 (2.7%)	447
5	*Clinical Oncology*	Oncology	3.0	28 (2.6%)	536
6	*Radiation Oncology*	Oncology	3.2	25 (2.3%)	916
7	*BMC Cancer*	Oncology	3.4	22 (2.1%)	1,226
8	*Clinical and Translational Radiation Oncology*	Oncology	2.7	16 (1.5%)	100
9	*Strahlentherapie und Onkologie*	Oncology	2.5	15 (1.4%)	208
10	*Seminars in Radiation Oncology*	Oncology	3.2	14 (1.3%)	276

The top 10 highest locally cited articles are listed in Table 5. Notably, the top two articles—SABR-COMET (13) and STOMP (14)—published in *The Lancet* and *Journal of Clinical Oncology* respectively, are pivotal clinical trials that have significantly shaped the understanding and practice of SABR for oligometastases.

**Table 5:** The top 10 highest locally cited articles.

Rank	Title (author)	Year	Journal	Local citations (within analyzed corpus)	Total citations	H-index
1	Stereotactic Ablative Radiotherapy Versus Standard of Care Palliative Treatment in Patients with Oligometastatic Cancers (SABR-COMET): a Randomized, Phase 2, Open-Label Trial (Palma DA)	2019	*Lancet*	294	1,473	20
2	Surveillance or Metastasis-Directed Therapy for Oligometastatic Prostate Cancer Recurrence: a Prospective, Randomized, Multicenter Phase II Trial (Ost P)	2018	*Journal of Clinical Oncology*	229	1,066	17
3	Characterization and Classification of Oligometastatic Disease: a European Society for Radiotherapy and Oncology and European Organization for Research and Treatment of Cancer Consensus Recommendation (Guckenberger M)	2020	*Lancet Oncology*	200	804	16
4	Stereotactic Ablative Radiotherapy for the Comprehensive Treatment of Oligometastatic Cancers: Long-Term Results of the SABR-COMET Phase II Randomized Trial (Palma DA)	2020	*Journal of Clinical Oncology*	195	935	20
5	Outcomes of Observation *Vs* Stereotactic Ablative Radiation for Oligometastatic Prostate Cancer the Oriole Phase 2 Randomized Clinical Trial (Phillips R)	2020	*JAMA Oncology*	174	884	9
6	Oligometastases Revisited (Weichselbaum RR)	2011	*Nature Reviews Clinical Oncology*	168	774	3
7	Defining Oligometastatic Disease from a Radiation Oncology Perspective: an ESTRO-ASTRO Consensus Document (Lievens Y)	2020	*Radiotherapy and Oncology*	142	492	13
8	Oligometastases Treated with Stereotactic Body Radiotherapy: Long-Term Follow-Up of Prospective Study (Milano MT)	2012	*International Journal of Radiation Oncology Biology Physics*	122	373	8
9	Stereotactic Body Radiotherapy for Oligometastases (Tree AC)	2013	*Lancet Oncology*	114	415	14
10	Stereotactic Body Radiotherapy for Multisite Extracranial Oligometastases Final Report of a Dose Escalation Trial in Patients with 1 to 5 Sites of Metastatic Disease (Salama JK)	2012	*Cancer*	102	282	16

### 3.5 Analysis of high-impact publications

**Figure 5:**
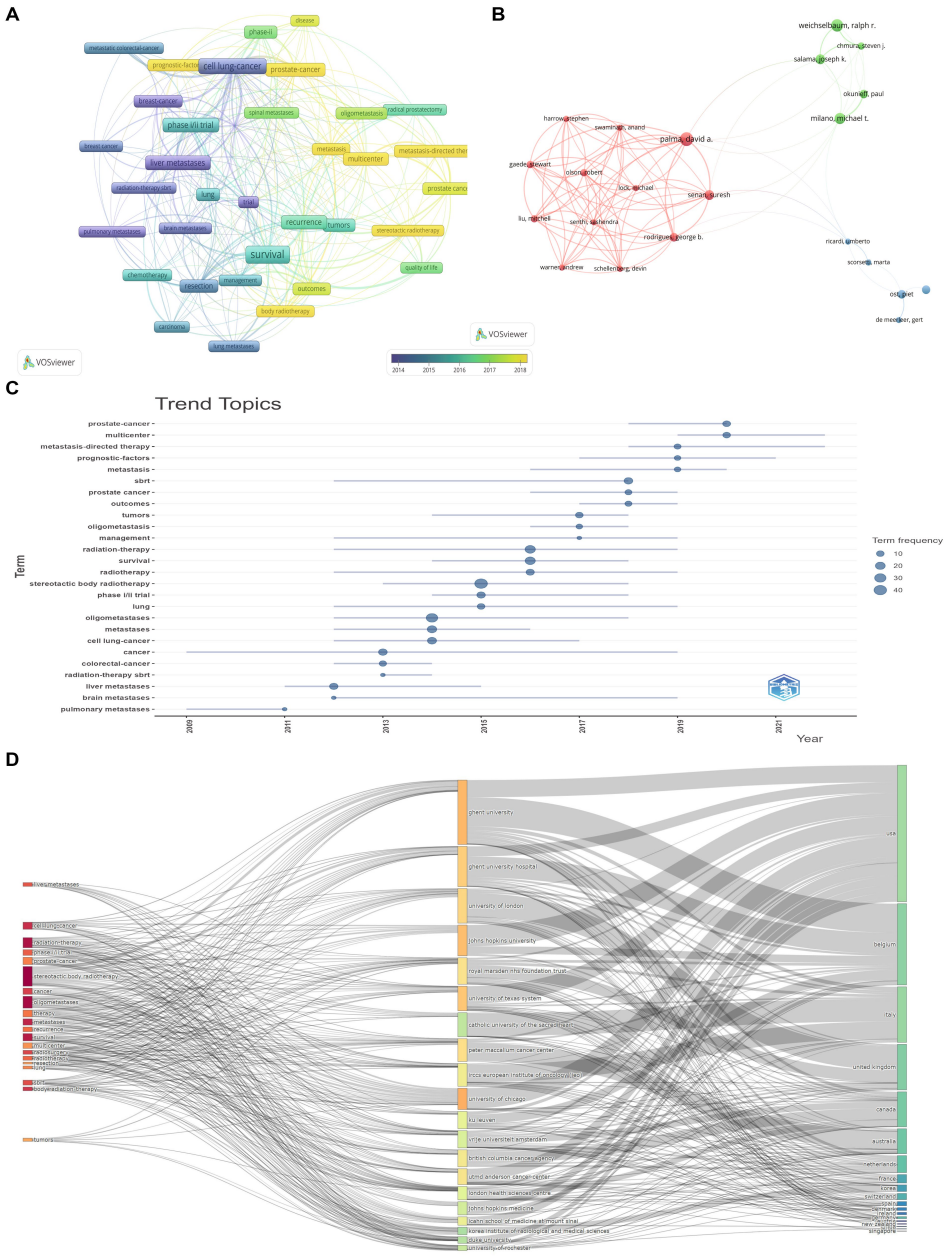
Analysis of the top 10% highly cited publications. **(A)** Keyword co-occurrence evolution network. Research trends have shifted from focusing on general cancer treatment and metastasis identification, towards precise, targeted therapies for metastatic diseases (e.g., metastasis-directed therapy, oligometastasis), and emphasizing a comprehensive evaluation of patient survival, quality of life, and prognostic factors. Multicenter clinical trials and research are also becoming increasingly important. **(B)** Co-authorship network. The figure clearly shows the author co-authorship network divided into three closely collaborating clusters, highlighting that researchers primarily engage in deep collaboration within their respective teams. **(C)** Trend topics evolution chart. **(D)** Alluvial diagram. This alluvial diagram visually depicts, through flow lines, how highly-cited research topics (particularly in cancer treatment and metastasis management) are channeled to specific contributing institutions, and subsequently attributed to their respective countries, thereby clearly revealing the global research landscape’s institutional expertise, collaborative networks, and national influence within these specialized areas.

A stratified analysis of highly cited publications emphasizes influential contributions to the field (Figure 5). As shown in Table 5 and further explored in Figure 5, the most highly cited articles primarily consist of clinical trials providing robust evidence for the efficacy and safety of SABR. The co-occurrence evolution network presented in Figure 5A clearly demonstrates that historical research primarily focused on basic metastasis identification, whereas recent trends heavily favor metastasis-directed therapy and long-term quality of life assessments. Figure 5B details the tight-knit scientific communities driving these landmark papers, revealing multi-institutional author clusters that dominate the citation landscape. Figure 5C tracks these specific trend topics over time, showing a sharp rise in “SABR safety and phase 2 validations” post-2018. Finally, the Alluvial Diagram in Figure 5D maps the direct flow of these highly cited paradigms to specific contributing institutions (such as University of Melbourne and Johns Hopkins) and their respective countries, confirming that early adoption of clinical trials by these specific hubs directly correlated with their current global influence in the oligometastases field.

## 4 Discussion

### 4.1 Global trends and conceptual evolution in SABR for oligometastases research

This bibliometric analysis unequivocally demonstrates robust and accelerating growth in research concerning SABR for oligometastases over the past two decades. This surge is reflected in the increasing annual publication volume and citation counts (Figure 2), signifying a maturing field with substantial academic interest and clinical impact. The global distribution of research, with the United States, Italy, and Canada as leading contributors (Table 1), highlights widespread recognition of SABR’s potential, as detailed in their co-authorship networks (Figure 3A). The prominence of specialized cancer centers and universities (Table 2) further underscores the interdisciplinary nature of this research, involving radiation oncology, medical oncology, and surgical specialties (Figure 3B).

Supplementary Figure 1 effectively illustrates how SABR has driven a “Paradigm Shift” in the treatment approach for oligometastatic cancers, decisively moving beyond the traditional “palliative-only” perspective. This transformation is characterized by a renewed focus on the potential for cure or long-term control and a strong emphasis on ablative local treatment (1, 2, 4, 15). Core “Modern SABR Features”—high-precision local ablation, non-invasive delivery, and synergy with systemic therapies—collectively underscore its role in offering aggressive strategies for limited metastatic disease (16).

The timeline in Supplementary Figure 1 details “Key advances in SABR for Oligometastases,” tracing its evolution from the foundational “Oligometastasis Hypothesis” (pre-2000) (17) to “Technical Maturation” (c. 2008) driven by 4D-CT and IGRT. Critical “Clinical Validation” arrived around 2017 with pivotal trials like SABR-COMET and ORIOLE (18, 19). More recently (c. 2020), the field entered an era of “Synergistic Integration,” characterized by the incorporation of AI-driven planning and SABR-immunotherapy combinations (16, 20). Looking forward to 2024 and beyond, trends point towards advanced “AI & Personalization,” indicating a dynamic future. This shift defines the contemporary landscape of oligometastases management.

### 4.2 Clinical evidence and emerging trials

The high citation counts for clinical trials (Table 5), discussed in Figure 5, underscore the field’s emphasis on evidence-based practice. The SABR-COMET trial, demonstrating improved survival with SABR, fundamentally altered clinical perceptions (21). Similarly, the ORIOLE trial provided robust data for SABR in oligometastatic prostate cancer (22). These studies have been instrumental in establishing the clinical utility of SABR.

Beyond established evidence, the field is actively exploring new indications through ongoing trials (Table 6). Phase II/III trials are currently recruiting for various sites, including breast cancer (NCT06135714), renal cell carcinoma (NCT06726421) (23–25), prostate cancer (NCT04787744) (26–28), and colorectal neoplasms (NCT06778382). These trials aim to evaluate SABR in combination with systemic therapies (16, 29), assess long-term outcomes, and explore SABR’s role in delaying systemic therapy (23). The ongoing “Stereotactic Radiotherapy for Oligometastasis (1–5) in Various Tumor Sites vs. Palliative Care” (NCT06556550) trial exemplifies efforts to broaden applications (30–32). These reflect a concerted effort to build a stronger evidence base for personalized strategies.

**Table 6:** SABR for oligometastases in phase 2 or 3 clinical trial.

Disease	Clinical trial	Phase	Study group	Primary outcome	Conclusions
Oligometastatic disease	NCT06556550: Stereotactic Radiotherapy for Oligometastasis (1–5) in Various Tumor Sites vs. Palliative Care	Phase 2/3	Patients with oligometastatic tumors (1–5 in bones/internal organs) aged >18 (*N* = 100) are compared between stereotactic radiotherapy and palliative care.	Progression-free survival, 2 years; Time before the current drug line change, 2 years	Recruiting; no results available yet. The study aims to increase the effectiveness of stereotactic radiation therapy for oligometastases compared with palliative therapy.
Metastatic breast cancer	NCT06135714: Metastasis-directed Therapy for Oligometastases of Breast Cancer (OLIGAMI)	Phase 3	Patients with breast cancer, oligometastasis, and metastatic breast cancer (*N* = 340) receive 12-week subtype-specific systemic therapy, then randomized to either continue systemic therapy (Arm A) or MDT + systemic therapy (Arm B).	Overall survival after second registration, 5 years	Recruiting; no results available yet. The trial aims to evaluate the efficacy of metastasis-directed therapy combined with systemic therapy for oligometastatic breast cancer.
Renal cell carcinoma metastatic	NCT06726421: Systemic Therapy Alone or with Stereotactic Body Radiotherapy for Oligometastatic Kidney Cancer (STROKER Study)	Phase 3	Randomized controlled trial evaluating SBRT efficacy in oligometastatic renal cell carcinoma (*N* = 252). Compares SBRT + systemic therapy vs. systemic therapy alone.	Progression free survival (PFS), up to 5 years	Recruiting; no results available yet. The study aims to determine if SBRT plus standard systemic therapy prolongs survival and impacts toxicity/QoL compared to standard systemic therapy alone.
Metastatic prostate cancer	NCT04787744: Veterans Affairs Seamless Phase II/III Randomized Trial of STAndard Systemic theRapy With or Without PET-directed Local Therapy for Oligometastatic pRosTate Cancer (VA STARPORT)	Phase 2/3	Veterans with oligometastatic prostate cancer (1–10 sites) (*N* = 464) randomized to standard systemic therapy with or without PET-directed local therapy (surgery or radiation).	Castration-resistant prostate cancer-free survival (CRPC-free survival), 4 years	Recruiting; no results available yet. The study aims to compare standard systemic therapy with or without PET-directed local therapy in improving CRPC-free survival for Veterans with oligometastatic prostate cancer.
Colorectal neoplasms malignant	NCT06778382: Delayed Systemic Therapy Following Destructive Local Treatment of Pulmonary Oligometastases After No Evidence of Disease (NED) in Colorectal Cancer.	Phase 2	Colorectal cancer patients with newly developed pulmonary oligometastases and NED after surgical resection (*N* = 22). Local treatment includes surgery, radiotherapy, or radiofrequency ablation.	Time Without Systemic Therapy, 2 years	Recruiting; no results available yet. The study aims to determine if systemic treatment should be added after local treatment for newly developed pulmonary oligometastases in colorectal cancer patients who have achieved No Evidence of Disease (NED).

### 4.3 Challenges encountered by SABR research

Despite promising advancements, several challenges persist within the landscape of SABR research for oligometastases. First, accurate patient selection remains complex; identifying which patients will benefit most requires sophisticated imaging and molecular profiling (17, 33, 34). Second, optimizing the integration of SABR with evolving systemic therapies (e.g., immunotherapy) is crucial, with questions regarding sequencing and toxicity requiring further investigation (16, 24). Third, while local control is high, distant progression remains a concern (5, 35). Finally, long-term follow-up data from large-scale randomized trials are needed to fully understand the impact on overall survival and quality of life (32, 36).

### 4.4 Future perspectives

The future of SABR for oligometastases is poised for innovation. Precision medicine will be central, utilizing advanced biomarkers and genomic profiling to refine patient selection (33, 37–39). The integration of Artificial Intelligence (AI) holds immense promise for enhancing treatment planning and dose optimization, potentially leading to fully adaptive radiotherapy (20, 40). As indicated in Figure 4C, AI aids in identifying optimal candidates and predicting outcomes. Furthermore, research will focus on combinatorial strategies with novel systemic therapies to enhance efficacy (e.g., abscopal effect) (24, 27, 29). Advanced imaging techniques (e.g., PET-CT) will improve target delineation (41, 42). Finally, large international registries will facilitate robust, multi-institutional trials to solidify SABR’s role in curative-intent management (43–46). Finally, large international registries will facilitate robust, multi-institutional trials to solidify SABR’s role in curative-intent management (47–50).

### 4.5 Limitations

While this bibliometric analysis provides a quantitative overview, it has limitations. First, data were retrieved exclusively from WoSCC and PubMed, potentially excluding relevant grey literature. Second, the study did not assess the quality of individual publications beyond citation counts, potentially treating all articles equally. Third, this is not a systematic review or meta-analysis; thus, it does not provide quantitative comparisons of therapeutic efficacy. Fourth, the restriction of search terms (e.g., “oligometasta*”) to the title field to increase specificity may have introduced a potential selection bias, possibly excluding studies focusing broadly on “metastasis-directed therapy” or clinical trials where SABR is central but terminology varies in the abstract. Findings should be interpreted as reflective of core research trends rather than absolutely exhaustive clinical evidence. Lastly, citation metrics may overlook recent advances that have not yet accumulated citations.

## 5 Conclusion

This comprehensive bibliometric analysis reveals a dynamic and accelerating research landscape for SABR in oligometastases. Since 2006, the field has experienced remarkable and consistent growth in both publication volume and citation impact, driven by significant contributions from leading nations (USA, Italy, Canada) and prolific institutions. Landmark clinical trials (e.g., SABR-COMET, ORIOLE) underscore the robust evidence base, establishing SABR as a transformative modality with curative potential. Key themes revolve around specific cancer types, metastatic sites, and synergistic integration with systemic therapies and AI.

Future endeavors are expected to increasingly focus on precision medicine by leveraging advanced biomarkers and genomic profiling for refined patient selection. The integration of AI promises to optimize treatment planning. Furthermore, extensive research into combinatorial strategies, alongside advancements in imaging and international collaborations, will be vital to further solidify SABR’s role in the curative-intent management of oligometastatic disease.

## Data Availability

The original contributions presented in the study are included in the article/Supplementary material, further inquiries can be directed to the corresponding authors.

## Supplementary material

SUPPLEMENTARY FIGURE 1 Key advances in SABR for oligometastases. This document illustrates how SABR has driven a paradigm shift in the treatment of oligometastatic cancer, challenging the traditional palliative view and evolving into a potentially curative modality, enabled by technological maturity, clinical validation, and deep integration with systemic therapies and AI.

SUPPLEMENTARY FIGURE 2 PRISMA-style flow diagram adapted for the current bibliometric study detailing the literature search, deduplication, and identification process.
