# Supplementary figures and images for: Bibliometric analysis of stereotactic ablative radiotherapy for oligometastases
Source: Front Med (Lausanne). 2026 Mar 18;13:1782986. doi: 10.3389/fmed.2026.1782986 (PMC13038553; doi:10.3389/fmed.2026.1782986)

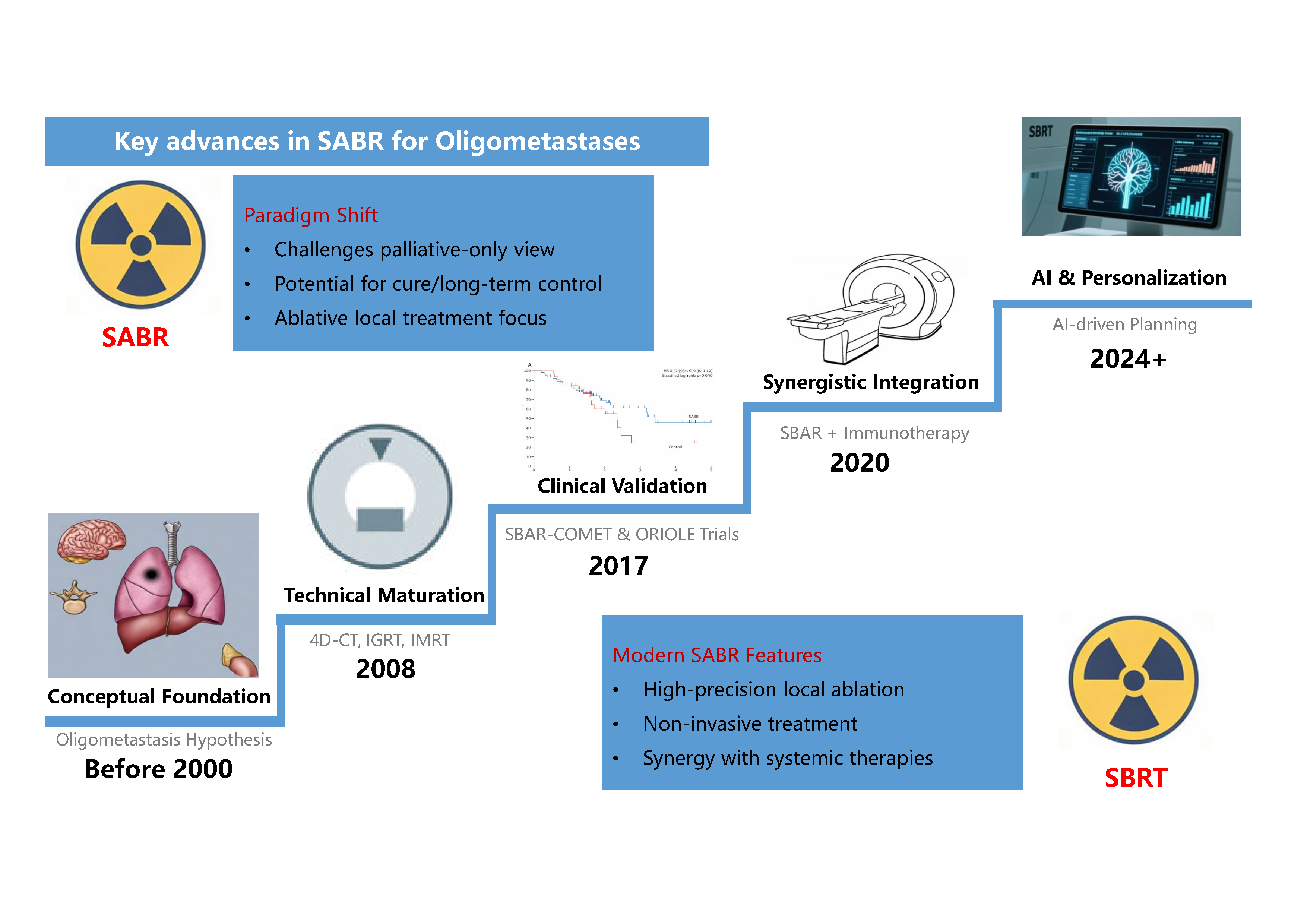

Supplement: SUPPLEMENTARY FIGURE 1 — Key advances in SABR for oligometastases. This document illustrates how SABR has driven a paradigm shift in the treatment of oligometastatic cancer, challenging the traditional palliative view and evolving into a potentially curative modality, enabled by technological maturity, clinical validation, and deep integration with systemic therapies and AI. [file Image_1.TIF]

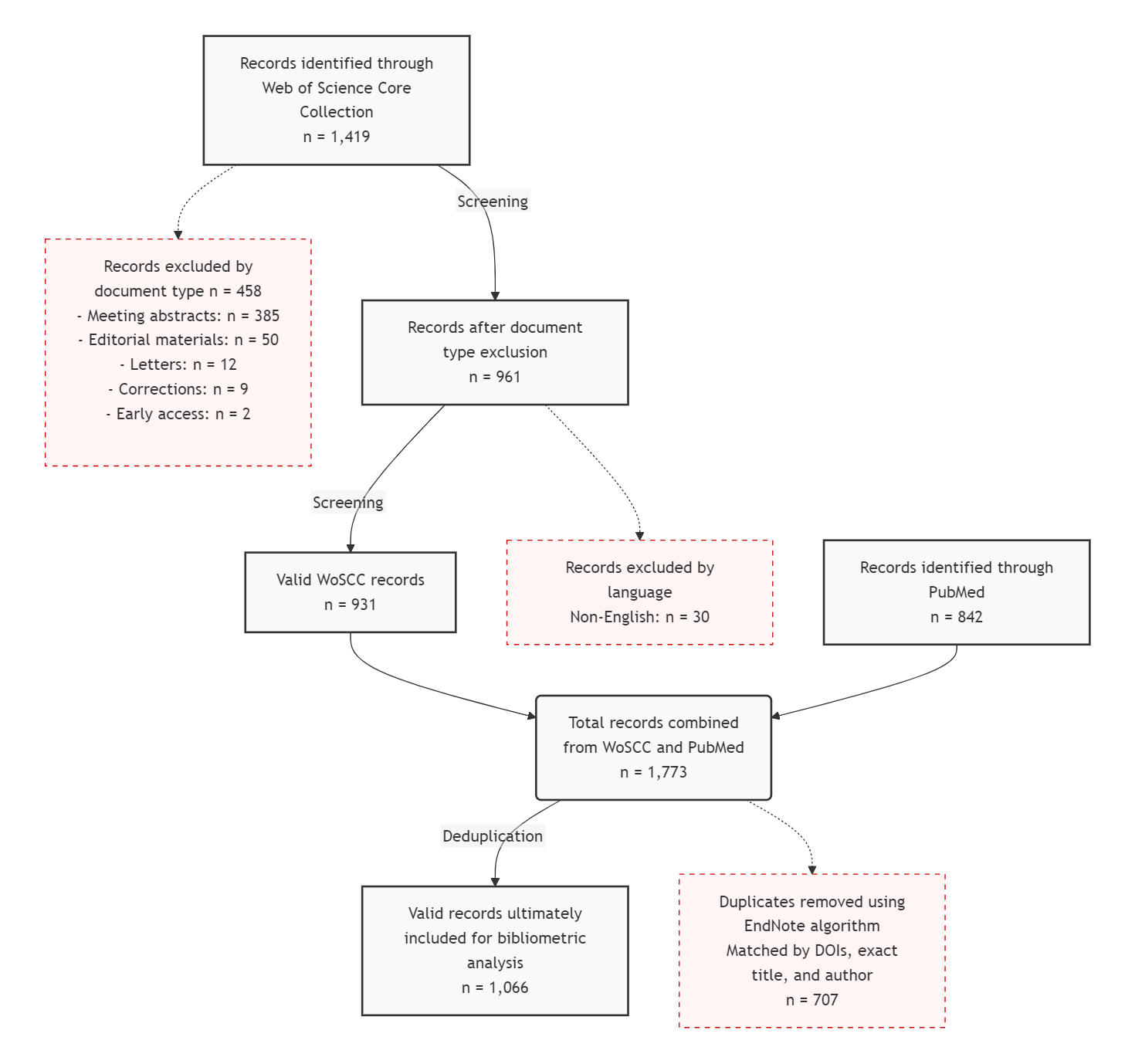

Supplement: SUPPLEMENTARY FIGURE 2 — PRISMA-style flow diagram adapted for the current bibliometric study detailing the literature search, deduplication, and identification process. [file Image_2.TIFF]
