# Supplementary material for: Bibliometric analysis of stereotactic ablative radiotherapy for oligometastases
Source: Front Med (Lausanne). 2026 Mar 18;13:1782986. doi: 10.3389/fmed.2026.1782986 (PMC13038553; doi:10.3389/fmed.2026.1782986)
Supplement: Supplementary file 3 [file Table_1.DOCX]

**Supplementary File 1: Full executable search strings utilized for WoSCC and PubMed databases.**

**Date of Search Execution:** January 5, 2026

**Timeframe Restriction:** January 1, 2006, to December 31, 2025

**Language Restriction:** English only

**Document Types Included:** Articles and Reviews (Meeting abstracts, editorial materials, letters, corrections, and early access records were excluded)

**1. Web of Science Core Collection (WoSCC)**

**Selected Indices:**
Science Citation Index Expanded (SCI-EXPANDED); Social Sciences Citation Index (SSCI)

**Building Blocks (Search Sets):**

- **#1 (Targeting primary disease):**
  TS=(“cancer*” OR “tumo*” OR “neoplas*” OR “carcinom*” OR “malign*”)
- **#2 (Targeting intervention):**
  TS=( ((“stereotactic body” OR “stereotactic ablative”) AND (“radiotherapy” OR “radiation” OR “radiation-therapy” OR “irradiation”)) OR “SBRT” OR “SABR” )
- **#3 (Targeting specific stage in Title):**
  TI=(“oligometasta*”)
  *(Note: This captures variations such as Oligometastasis, Oligometastases, and Oligometastatic)*

**Final Executable Search String for WoSCC (#1 AND #2 AND #3):**

(TI=“oligometasta*”) AND TS=("cancer*" OR "tumo*" OR "neoplas*" OR "carcinom*" OR "malign*") AND TS=( (( “stereotactic body” OR “stereotactic ablative”) AND (“radiotherapy” OR “radiation” OR “radiation-therapy” OR “irradiation” )) OR “SBRT” OR “SABR” )

**2. PubMed**

**Search Strategy Concept:**

Similar logic to WoSCC, utilizing [Title/Abstract] for searching topic-equivalent fields, and restricting “oligometasta*” strictly to the [Title] field to maintain high specificity and align with the WoSCC methodology.

**Final Executable Search String for PubMed:**

(oligometasta*[Title]) AND (cancer*[Title/Abstract] OR tumo*[Title/Abstract] OR neoplas*[Title/Abstract] OR carcinom*[Title/Abstract] OR malign*[Title/Abstract]) AND ("stereotactic body"[Title/Abstract] OR "stereotactic ablative"[Title/Abstract] OR SBRT[Title/Abstract] OR SABR[Title/Abstract])

*Note: The raw records downloaded from the above executable strings were subsequently integrated and deduplicated using EndNote software (based on primary unique DOIs, followed by strict title and author matching) prior to the final bibliometric analysis.*
